# High dissolved oxygen tension triggers outer membrane vesicle formation by *Neisseria meningitidis*

**DOI:** 10.1186/s12934-018-1007-7

**Published:** 2018-10-03

**Authors:** Matthias J. H. Gerritzen, Ronald H. W. Maas, Jan van den Ijssel, Lonneke van Keulen, Dirk E. Martens, René H. Wijffels, Michiel Stork

**Affiliations:** 1 Process Development Bacterial Vaccines, Institute for Translational Vaccinology (Intravacc), P.O. Box 450, 3720 AL Bilthoven, The Netherlands; 2 Bioprocess Engineering, Wageningen University, P.O. Box 16, 6700 AA Wageningen, The Netherlands; 3 Faculty of Biosciences and Aquaculture, Nord University, P.O. Box 1409, 8049 Bodø, Norway

**Keywords:** Outer membrane vesicles, *Neisseria meningitidis*, Oxidative stress, Dissolved oxygen changestat, Accelerostat

## Abstract

**Background:**

Outer membrane vesicles (OMVs) are nanoparticles released by Gram-negative bacteria and can be used as vaccines. Often, detergents are used to promote release of OMVs and to remove the toxic lipopolysaccharides. Lipopolysaccharides can be detoxified by genetic modification such that vesicles spontaneously produced by bacteria can be directly used as vaccines. The use of spontaneous OMVs has the advantage that no separate extraction step is required in the purification process. However, the productivity of spontaneous OMVs by bacteria at optimal growth conditions is low. One of many methods for increasing OMV formation is to reduce the linkage of the outer membrane to the peptidoglycan layer by knocking out the *rmpM* gene. A previous study showed that for *Neisseria meningitidis* this resulted in release of more OMVs. Furthermore, cysteine depletion was found to trigger OMV release and at the same time cause reduced growth and oxidative stress responses. Here we study the effect of growth rate and oxidative stress on OMV release.

**Results:**

First, we identified using chemostat and accelerostat cultures of *N. meningitidis* that increasing the growth rate from 0.03 to 0.18 h^−1^ has a limited effect on OMV productivity. Thus, we hypothesized that oxidative stress is the trigger for OMV release and that oxidative stress can be introduced directly by increasing the dissolved oxygen tension of bacterial cultures. Slowly increasing oxygen concentrations in a *N. meningitidis* changestat showed that an increase from 30 to 150% air saturation improved OMV productivity four-fold. Batch cultures controlled at 100% air saturation increased OMV productivity three-fold over batch cultures controlled at 30% air saturation.

**Conclusion:**

Increased dissolved oxygen tension induces the release of outer membrane vesicles in *N. meningitidis* cultures. Since oxygen concentration is a well-controlled process parameter of bacterial cultures, this trigger can be applied as a convenient process parameter to induce OMV release in bacterial cultures. Improved productivity of OMVs not only improves the production costs of OMVs as vaccines, it also facilitates the use of OMVs as adjuvants, enzyme carriers, or cell-specific drug delivery vehicles.

**Electronic supplementary material:**

The online version of this article (10.1186/s12934-018-1007-7) contains supplementary material, which is available to authorized users.

## Background

Outer membrane vesicles (OMVs) are naturally produced by Gram-negative bacteria and play a role in pathogenesis, cell-to-cell communication and stress responses [1]. OMVs are spherical nanoparticles that consist of a phospholipid bilayer with proteins and lipopolysaccharides (LPS) [1]. The lumen of the vesicle contains DNA and periplasmic components of the bacterium [2, 3]. Membrane vesicle formation has been shown recently in Gram-positive bacteria and archaea as well [4, 5].

OMVs are highly similar to the outer membrane of the bacteria, are non-replicating, and characteristically are full of pathogen associated molecular patterns. With this they fulfill major criteria for vaccine design and have been successfully used as such [6, 7]. These vaccines have been produced by extraction of vesicles from the bacterial outer membrane using detergents. In this way, vesicles are artificially formed and the amount of toxic LPS could be reduced [8, 9]. However, extraction of vesicles is disadvantageous since the proteome of extracted OMVs (eOMVs) shows a lowered amount of possible immunogenic proteins over spontaneously released OMVs (sOMVs) [10, 11]. Furthermore, extraction methods are not required anymore for LPS removal since the introduction of genetically modified low toxicity LPS [12, 13], which forms the basis for the use of spontaneously released OMV. Thus, the use of spontaneous released vesicles simplifies the purification of OMVs since it obsoletes the extraction step in the down-stream processing of the vaccine product [10, 11]. Sera from mice immunized with spontaneous OMVs show immunity against a broader range of serotypes than mice immunized with detergent extracted OMVs [14]. Furthermore, omission of detergent also preserves vesicle integrity, yielding a more uniform vaccine product.

Feasible sOMV production has not been straightforward since sOMV productivity at optimal growth conditions is low. Despite the research on OMV biogenesis over the past 4 decades, the exact mechanism triggering the release of OMVs by a bacterium remains unknown. Because the composition of OMVs differs from the outer membrane of the bacteria, it is generally thought that the release of vesicles is not a random process [15]. Biogenesis of OMVs has been described by several models although it remains unclear whether a shared mechanism exists [16]. OMV biogenesis is hypothesized to be based on peptidoglycan fragments accumulation in the periplasm, less anchoring of the outer membrane to the peptidoglycan layer, or O-antigen charge repulsion. These models are reviewed in [17] and [2]. Recently, Roier et al. suggested a novel mechanism based on phospholipid accumulation that is conserved among Gram-negative bacteria [18]. The proposed phospholipid transporter VacJ/Yrb ATP-binding cassette was shown to be involved in OMV production and could be part of regulated OMV release. Increasing the OMV production by *Neisseria meningitidis* is possible by deleting the *rmpM* gene, the product of which anchors the outer membrane to the peptidoglycan layer [14]. Reducing the linkage between the outer membrane and the peptidoglycan layer results in so-called blebbing mutants of bacteria that show increased release of OMVs in the supernatant. This was found not only for *N. meningitidis*, but also for *E. coli* [19].

External triggers for OMV release could be a convenient way to enhance production in bioreactor cultures. Van de Waterbeemd et al. showed that cysteine depletion can be used as a trigger to stimulate the release of vesicles in *N. meningitidis* cultures [20]. Simultaneously with cysteine depletion, the growth rate is reduced and oxidative stress responses were observed in the transcriptome of the bacterium. It is unknown whether cysteine directly triggers OMV formation or works indirectly through a reduction in growth rate and/or increase in oxidative stress. Furthermore, increased release of vesicles under hydrogen peroxide addition has been shown [20]. The method of hydrogen peroxide addition, however, is not feasible for scalable production processes of OMV since local hydrogen peroxide addition to a bacterial culture will result in significant cell death and lysis. In this study we hypothesize that extracellular oxidative stress is directly induced by high concentrations of dissolved oxygen, which is one of the controlled parameters in bioreactor cultivations. The dissolved oxygen tension is typically kept low, to minimize the stress from hyperoxia and to prevent oxygen inhibition [21]. Especially for a facultative anaerobic pathogen it is obvious to design the cultivation with low oxygen concentration [22]. For example, *N. meningitidis* cultivation for both the vaccine concepts HexaMen and NonaMen has been designed with levels of 30% air saturation [23, 24].

The aim of this study is to obtain more insight in the role of growth rate and oxidative stress in the release of OMVs. The first section of this paper will examine whether a decrease in growth rate can trigger OMV release by using accelerostat experiments. Next, oxidative stress is introduced in continuous cultures by increasing the dissolved oxygen tension. Lastly, increasing the dissolved oxygen concentration is tested on batch cultures.

## Results

### *Neisseria meningitidis s*OMV release at reduced growth rate

**Fig. 1:**
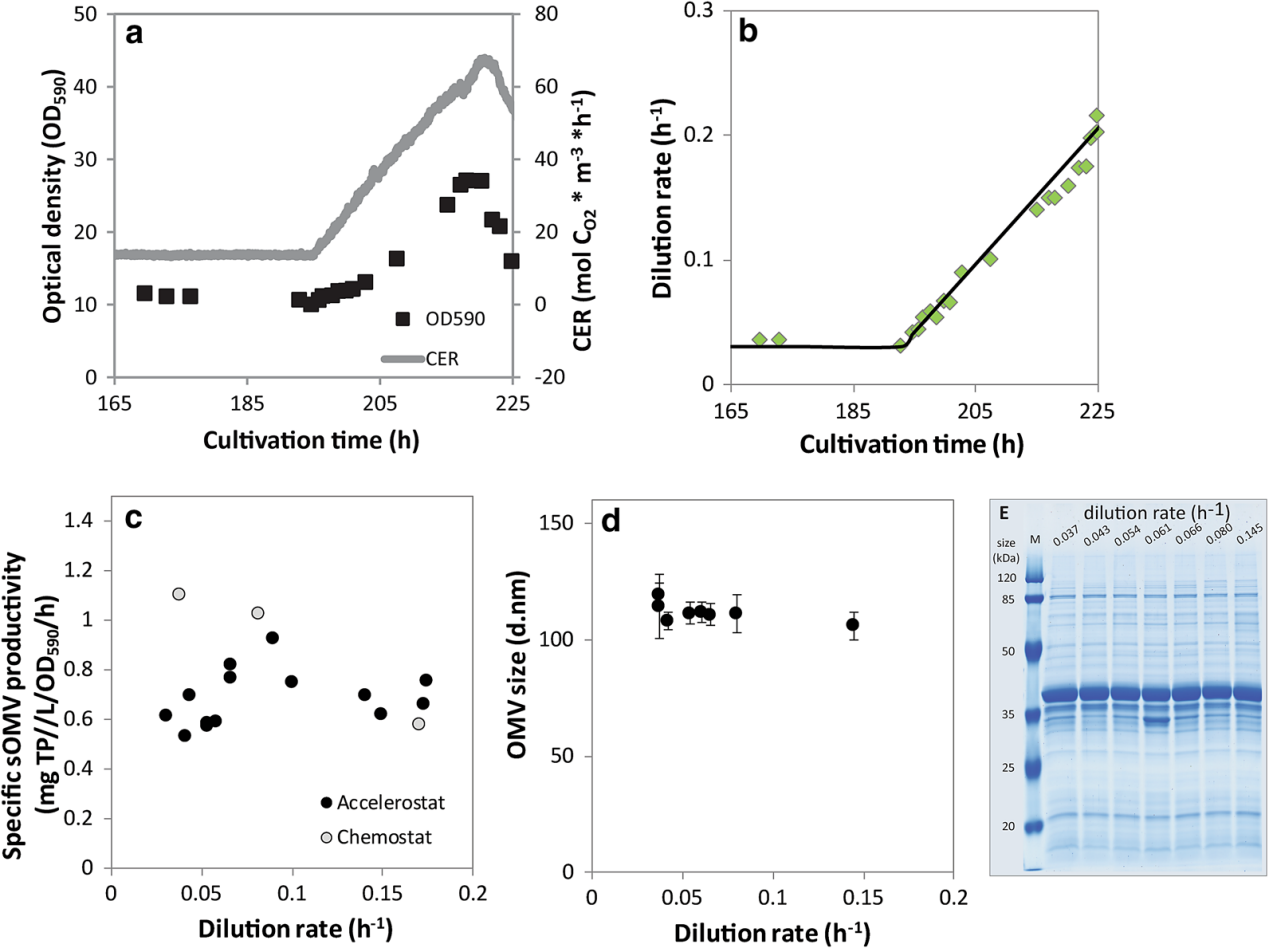
Influence of the growth rate on OMV release in a *N. meningitidis* accelerostat. **a** The optical density (black squares) and the carbon dioxide evolution rate of the accelerostat culture (grey line). **b** The increase of the dilution rate (black line, *a*_*D*_ of 0.0055 h^−2^), the actual measured dilution rate (diamonds). **c** The resulting specific OMV productivity (mg of total protein (TP) per liter culture of OD_590_ per hour ) at different dilution rates for the accelerostat (solid circles) and chemostats (open circles). Vesicles were purified from the accelerostat at different dilution rates and the size of the purified OMVs is shown in **d**. Error bars represent the standard deviation of the measurement. The protein composition of the OMVs is analyzed by SDS-PAGE (**e**). Lane 1 contains a molecular weight marker and lane 2–8 contain sOMVs purified at increasing dilution rates

The increased productivity of OMVs during the stationary phase of a batch cultivation [20] raised the question what the direct influence of the growth rate on the sOMV release was. Here we assessed the influence of growth rate on sOMV release in three chemostat cultures in steady state at different dilution rates, and in an accelerostat, by slowly increasing the dilution rate of a chemostat culture of *N. meningitidis*. The slow change in dilution rate (*a*_*D*_) should keep the culture in steady state in this approach [25]. In this accelerostat an acceleration rate (*a*_*D*_*)* of 0.0055 h^−2^ was used (Fig. 1b). The carbon dioxide evolution rate (CER) and the optical density increased simultaneously with the dilution rate (Fig. 1a). In the accelerostat, OMVs were produced during the whole culture and were also similar in size and protein composition throughout the culture (Fig. 1c, d). The specific sOMV production was constant throughout the culture with the growth rate ranging from 0.03 to 0.18 h^−1^ (Fig. 1b). Chemostat cultures at three different growth rates showed comparable productivity to the accelerostat at a growth rate of 0.18 h^−1^, while the two lower growth rates show a minor increase in OMV productivity. From these results, we conclude that reducing the growth rate from 0.18 to 0.03 h^−1^ is not an important trigger for sOMV release.

### Influence of oxidative stress in a dissolved oxygen tension changestat

**Fig. 2:**
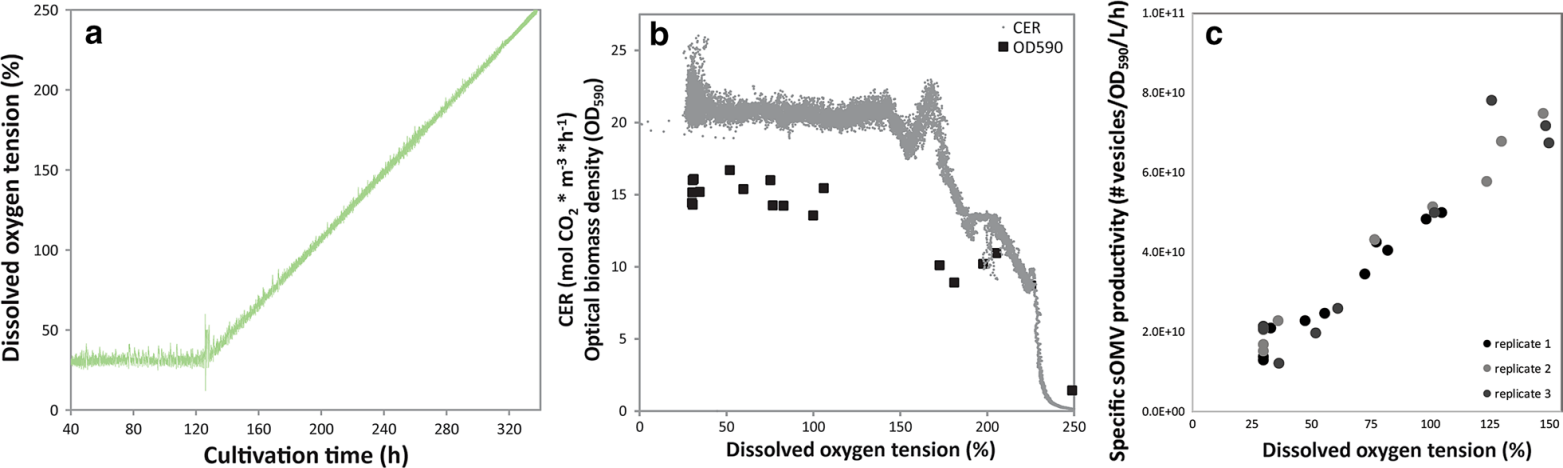
The influence of increased dissolved oxygen tension on growth and OMV productivity. **a** The control of the dissolved oxygen concentration in the changestat, where the dissolved oxygen tension was increased by 1% per hour. The effect of the elevated oxygen concentration on the growth is shown in **b**. The carbon dioxide evolution rate (grey) and bacterial biomass density (black squares) is similar for oxygen concentrations up to 150% air saturation. **c** The specific productivity of sOMVs as a function of DO for three replicate cultures. The results for replicate 1 as shown in **a**, **b** are representative for the two other replicates

The effect of increased oxygen concentration on bacterial growth and OMV release was assessed with a changestat approach. The dissolved oxygen tension of a chemostat culture is linearly increased at a rate (1%/h) that should be sufficient low to maintain a steady state culture (Fig. 2a). *N. meningitidis* is capable of growth up to 150% air saturation without significant impact on the carbon dioxide evolution rate (Fig. 2b). The release of sOMVs is linearly linked to the concentration of oxygen in the culture broth (Fig. 2c). sOMV production can be increased by a factor 4 at high oxygen concentration, while preserving growth of the bacteria. Between dissolved oxygen concentrations of 150% and 220% air saturation, bacterial growth is affected, illustrated by the declined carbon dioxide production rate and lower biomass concentration (Fig. 2b). Production of OMVs at these levels is not preferred.

**Fig. 3:**
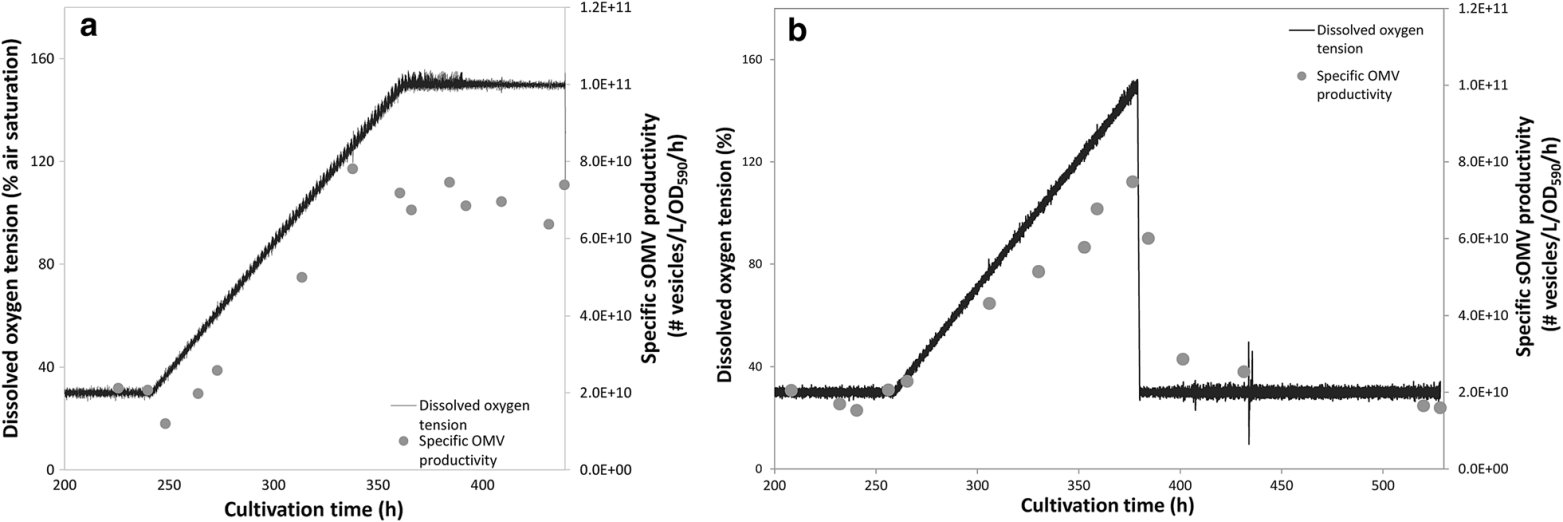
Verification of the dissolved oxygen changestat cultures. One replicate of the *N. meningitidis* dissolved oxygen changestat was maintained at 150% air saturation upon reaching this value (**a**). The specific sOMV productivity remained at 7 × 10^10^ sOMVs/L culture OD_590_ per hour , confirming the measurements in the changestats. Another replicate was returned to a steady state at 30% air saturation (**b**). During 5 dilutions, wash out of the OMVs produced at increased oxygen concentrations during the changestat was observed, resulting in a steady with similar productivity (1.6 × 10^10^ sOMVs/L culture OD_590_ per hour ) to the beginning of the changestat (1.7 × 10^10^ sOMVs/L culture OD_590_ per hour)

Next, the changestat was repeated until a dissolved oxygen concentration of 150% after which the setpoint was maintained constant at this value. The culture showed a steady state productivity at a similar level as at the corresponding oxygen concentration during the changestat (Fig. 3a), confirming that the accelerating factor of the changestat was sufficiently low to keep the culture in steady state. Last, a third changestat culture was done where after reaching a dissolved oxygen concentration of 150% the setpoint was returned to the starting value of 30%. The specific productivity returned to the level at the start of the changestats indicating that the changestat culture did not induce changes to the bacteria (Fig. 3b). Furthermore, the increased oxygen concentrations also showed to trigger sOMV release in an *E. coli* dissolved oxygen changestat (Additional file 1: Figure S1). In summary, it is shown that increased oxygen tension triggers OMV formation.

### Improved productivity of batch cultures at increased oxygen concentrations

**Fig. 4:**
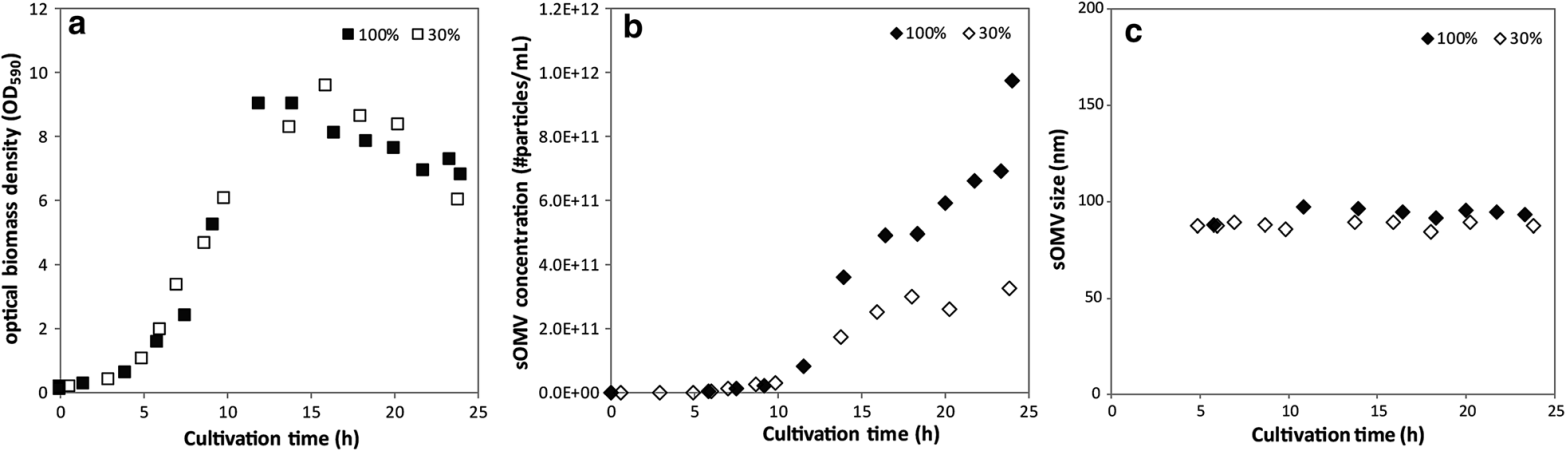
High dissolved oxygen tension induces OMV release in *N. meningitidis* batch cultures. Growth curves of *N. meningitidis* batch cultures controlled at 30% (open symbols) and 100% air saturation (solid symbols) show similar growth (**a**). The increased oxygen concentration showed to induce a higher level of vesicle release (**b**). Graphs are the overlay of two replicate cultures to practically allow for sufficient data points covering 24 h. The first replicate consists of data points at 0 h to 12 h cultivation and at 24 h cultivation, and the second replicate at 0 h and 15 h to 22 h. **c** The mode size of sOMV particles as measured by NTA in the supernatants from a *N. meningitidis* batch cultivation at two dissolved oxygen concentrations (30% air saturation and 100% air saturation)

The high dissolved oxygen concentration was applied to a *N. meningitidis* batch cultivation to assess the feasibility of increased sOMV production. A dissolved oxygen tension of 100% air saturation was used since this value showed increased OMV release while maintaining similar growth characteristics as at 30% air saturation in the changestat (Fig. 2b). Bacteria were grown in chemically defined medium that results in sOMV release from the onset of the stationary phase. The bacterial growth profile was similar for the batch cultures at 30% and 100% air saturation, showing the capability of *N. meningitidis* to deal with higher oxygen concentrations (Fig. 4a). The higher oxygen concentration triggered an increased release of vesicles resulting in a three-fold higher productivity at the end of the culture compared to the standard level of 30% (Fig. 4b). The size of OMVs remains constant throughout the culture and is similar between the two oxygen concentrations (Fig. 4c). High dissolved oxygen levels are therefore a convenient method for increasing sOMV production in batch cultures.

## Discussion

In this study, we investigated reduced growth rate and oxidative stress as triggers to induce sOMV formation in *N. meningitidis*. In the accelerostat experiment, the growth rate increases linearly with the CER up to a growth rate of 0.18 h^−1^. At higher dilution rates, a reduction in CER was observed and the experiment was stopped. The maximum specific growth rate of *N. meningitidis* on this medium is 0.5 h^−1^ [14] and wash-out is thus not expected at this dilution rate. A change in limiting substrate could explain the results at growth rates over 0.18 h^−1^. All chemostats showed depletion of the carbon sources glucose and glutamate and the cultures were likely carbon limited. A lowered bacterial density was observed at reduced bacterial growth rates, that could be explained by the increased energy requirement for maintenance. The biomass yield on substrate (Y_xs_) and the maintenance coefficient (m_s_) were 0.43 g_biomass_.g_glucose_^−1^ and 0.08 g_glucose_.g_biomass_^−1^ h^−1^, calculated using the maintenance model of Pirt [26]. These values were in line to the values of aerobic glucose limited chemostat cultures reported by Baart et al. (0.44 g_biomass_.g_glucose_^−1^ and 0.04 g_glucose_.g_biomass_^−1^ h^−1^ for resp. Y_xs_ and m_s_) [27]. For the chemostats there seems to be an increase in specific productivity when the growth rate is lowered, where the point at the lowest growth rate significantly deviates from the accelerostat measurement. Since the lowest growth rate is the starting point of the accelerostat, which is certainly in steady state, this difference is not due to a too high acceleration rate in the accelerostat. Together, this data shows that reducing the growth rate from 0.18 to 0.03 h^−1^ does not have a large effect on OMV productivity, although due to the contrasting results it is not clear if a minor increase in productivity is associated with lowered growth rates.

The effect of oxidative stress was assessed by changestat cultures with increasing dissolved oxygen tensions. *N. meningitidis* showed to be capable of handling dissolved oxygen concentrations of up to 220% air saturation. OMV productivity was increased fourfold in a changestat culture at oxygen concentrations elevated to 150% air saturation and threefold in a batch culture controlled at 100% air saturation in comparison to cultures at 30% air saturation. Applying increased dissolved oxygen tension on *E. coli* resulted in a similar increase in sOMV release. The production of sOMVs by oxidative stress could be triggered in the bioreactor by controlling the oxygen concentration in the culture broth, although the exact route of OMV induction remains to be elucidated. Oxidative stress triggers sOMV release on top of the known genetic mutations that increase OMV formation [14, 19, 28]. These mutations reduce the linkage between the outer membrane and the peptidoglycan layer. Here we show the effect of oxidative stress on a *rmpM* knockout strain of *N. meningitidis* and on a Tol-Pal mutant strain of *E. coli.* Oxidative stress may be a general mechanism to induce sOMV release. Applying increased oxygen concentrations on a batch culture showed enhanced release of OMVs of similar size to OMVs produced in a batch culture with sulfur depletion alone, which is an indication that oxidative stress is the underlying trigger in OMV release triggered by sulfur depletion. Moreover, Sabra et al. showed by electron micrographs that *Pseudomonas aeruginosa* seldom forms membrane vesicles under anoxic conditions (~ 0% of air saturation), while under extreme oxidative stress conditions (350% of air saturation) membrane vesicles were observed [29]. Biologically *Neisseria* spp. encounter oxidative stress upon oxidative bursts of phagocytes [30, 31]. Lappann et al. showed that OMVs of *N. meningitidis* serve as a decoy for the bacteria to circumvent binding of the bacteria to neutrophil extracellular traps (NETs) by binding of OMVs to the NETs [32]. The response of forming OMVs by the bacterium could thus enhance bacterial survival by avoiding phagocytosis and NET‐mediated killing. During infection, sOMV release probably contributes to disease progression and the severity of fulminant meningococcal sepsis [33, 34]. The biological role of OMVs in the interaction with phagocytes should gain more interest. Another explanation of OMV release under oxidative stress conditions would be that the OMVs alleviates stress of the bacterium. This method of stress release could be in the form of eliminating misfolded and unfolded proteins, as shown for *Pseudomonas aeruginosa* [35]. For *E. coli*, OMV formation has been shown advantageous to bacterial survival as response to periplasmic protein accumulation, and periplasmic peptidoglycan and LPS fragment accumulation was found to be associated with increased OMV release [36, 37]. Increased OMV release as response to increased oxidative stress could be advantageous to the bacterium in a similar manner.

During exponential growth in the batch culture, only minor OMV production is observed and high dissolved oxygen tension does not induce OMV release in this phase. A probable explanation is that *N. meningitidis* can handle the increased oxygen concentrations by their metabolism during unlimited growth. Production of oxidative stress is a characteristic of aerobic bacterial growth as components of the respiratory chain are oxidized [38]. *Neisseria* spp. are oxidase positive pathogens containing a mitochondrial like respiratory chain [39] and typically show high levels of respiration [40]. The *N. meningitidis* genome encodes multiple small c-type cytochromes and a single terminal cytochrome oxidase of the cbb3 type [41–44]. Li et al. hypothesized that the high respiratory capacity of *Neisseria* spp. and the excess capacity for oxygen reduction acts as defense against endogenous reactive oxygen species (ROS) [43]. SodA and MntC are the major effectors involved in the *Neisseria* spp. oxidative stress response [45, 46]. Upon cysteine depletion in batch cultures, high oxygen concentrations enhance the production of sOMVs. This increased release may be caused by a reduction in capacity of handling oxidative stress by the oxidative stress defense mechanisms due to cysteine limitation.

Our initial results show that OMV size was not affected although oxidative stress can cause damage to bacteria. In general, increased oxygen concentrations could affect bacterial growth and the production of biological compounds [47], as was observed in the changestat culture at concentrations over 150% air saturation. *Neisseria* spp. are adapted to ROS production, since reactive oxygen species accumulate as byproducts of the aerobic respiration [48, 49]. They thus contain several methods to handle ROS [42, 45]. The changestat experiments showed that increased oxygen concentrations can be controlled such that growth remains possible. Future work should ensure the quality of OMVs produced under oxidative stress remains consistent. Promising applications such as the additions and stabilization of enzymes on OMVs [50–52], the study of proteins in their native membrane environment [53], or the delivery of drugs packed in OMVs to specific cells [54] could also benefit from this production method.

## Conclusion

In summary, this study shows that the dissolved oxygen tension of *N. meningitidis* cultivations could be used to stimulate OMV release by the introduction of oxidative stress. Increasing the dissolved oxygen concentration of batch cultures from 30 to 150% resulted in a factor 4 increased specific productivity. The dissolved oxygen tension is a well-controlled process parameter to induce outer membrane vesicle formation. With this approach, OMV production can be improved reducing the production costs of OMV-based vaccines and facilitating the use of OMVs for other applications.

## Methods

### Bacterial strains

A recombinant derivate of the *Neisseria meningitidis* serogroup B isolate H44/76 [55] was used in this study. The selected strain was a PorA lacking derivate of the H44/76 isolate [56]. This strain has a non-encapsulated phenotype due to the *siaD* knockout, *lpxL1* deletion to attenuate LPS-toxicity, *rmpM* deletion to improve vesicle formation (unless indicated otherwise) and *lgtB* mutation to promote interactions with dendritic cells [14, 57]. This strain was stored in glycerol as working seedlots. All cultivations were performed in chemically defined growth medium [27].

For the cultivation with *Escherichia coli* strain JC8031 (TolRA) was used [58]. A shaker flask culture was started by adding 10 µL of frozen glycerol stock (− 80 °C) to 100 mL LB medium (Large Capsules: tryptone 10 g/L, yeast extract 5 g/L, NaCl 10 g/L, MP Biomedicals) and incubating the shaker flask at 37 °C for 16 h. Bioreactor cultivations were performed on LB medium without antifoam with a maximum stirrer speed of 600 RPM at 37 °C.

### Bioreactor cultivations

Batch cultivations were performed in 5 L dished bottom Applikon bioreactors with an H/D ratio of 1.6 based on total volume. Cultivations were operated with 3 L working volume on a Pierre Guerin Tryton^i^ controller. Temperature was controlled at 35 ± 0.5 °C and pH was controlled at pH 7.2 ± 0.05 using 1 M HCl and 1 M NaOH. Dissolved oxygen tension was controlled at 30% unless indicated otherwise. The membrane covered polarographic oxygen sensor (InPro 6850i, Mettler Toledo) was calibrated at 100% in air-saturated sterile growth medium of 35 °C. In the first phase of the cultivation, the dissolved oxygen tension is controlled by increasing the agitation rate (300–1000 RPM) followed by increasing the fraction of oxygen in the headspace aeration (1 L/min) by the addition of pure oxygen. The agitation rate of the 100% air saturation cultures was fixed at 1000 RPM directly after inoculation after which the oxygen tension was controlled by the addition of pure oxygen in the headspace. Samples were taken for optical density measurements and used for nutrient and sOMV measurements after sterile filtration (0.22 µm pore-size) and storage at 4 °C. Off-gas composition was analyzed by a Thermo Prima δb process mass spectrometer.

### Chemostat cultivations

Continuous cultivations were performed in a similar setup as the batch cultivation setup. The working volume of the 5 L bioreactor was decreased from 3.0 to 2.0 L to reduce the feed medium required for the experiments. The vessel was equipped with a medium inlet and two outlet pipes, one submerged in the cultivation broth at the height of the stirrer and one directly at the liquid–gas interphase. The latter allowed the control of the working volume to be exactly 2.0 L at a fixed maximum stirrer speed, independent of foaming. The weight of the bioreactor, the feed medium and the pH titrant solutions was measured by balances and used for verification of the dilution rate. Samples were taken for optical density measurements and off-gas analysis was similar to the batch cultivation. The bioreactor was controlled with the same control loops as used in the batch cultivations. After 8 h of growth the feed and the bleed pumps were started to initiate a continuous culture. Steady state of the culture was assumed based on stable bacterial density values and stable carbon dioxide emission for at least 3 dilutions of the bioreactor volume.

### Accelerostat and dissolved oxygen changestat cultivation

An accelerostat was started from a chemostat fermentation in steady state at D = 0.03 h^−1^, operated as described in the previous section, by increasing the dilution rate linearly with a_D_ = 0.0055 h^−2^. The dilution rate was changed by increasing the medium inflow rate and equally increasing the broth outflow rate. From the culture broth, 50 mL samples were drawn to purify sOMVs. The samples were centrifuged at 4000×*g* for 30 min at 4 °C and the sterile filtered supernatants (Nalgene RapidFlow 0.2 µm pore-size PES filter unit) were concentrated on 100 kDa cut-off spin filters. The concentrated sOMVs were washed with 3% sucrose buffered by 10 mM TrisHCl (pH 7.4) to wash out contaminating proteins. Next, the diafiltrated sOMVs were centrifuged at 125.000×*g* for 2 h. The sOMV containing pellet was dissolved in 1 mL 10 mM TrisHCl (pH 7.4) with 3% sucrose.

The dissolved oxygen tension changestat was started from a chemostat culture. For this, a continuous culture in steady state with µ = 0.04 h^−1^ was obtained as described previously. During this steady state, the oxygen concentration was controlled at 30% air saturation, the starting point for the changestat. From the start of the changestat, the oxygen concentration was increased linearly with a_DOT_ = 1.0%/h.

*Escherichia coli* JC8031 (TolRA) was used for the dissolved oxygen tension changestat of *E. coli* [58]. A shaker flask culture was started by adding 10 µL of frozen glycerol stock (− 80 °C) to 100 mL LB medium (Large Capsules: tryptone 10 g/L, yeast extract 5 g/L, NaCl 10 g/L, MP Biomedicals) and incubating the shaker flask at 37 °C for 16 h. Bioreactor cultivations were performed on LB medium without antifoam with a maximum stirrer speed of 600 RPM at 37 °C.

### Quantification of sOMVs

Culture samples were sterile filtered (0.22 µm pore-size) before the sOMV were measured. sOMV concentration was measured with a phospholipid specific probe FM 4-64 (SynaptoRed C2, Biotium) by mixing 50 µL of 2- to 50-fold diluted samples with 50 µL of dye solution (0.05 mM FM 4-64). Fluorescence was measured directly after mixing this solution using a plate fluorometer (Synergy MX, Biotek ex480, em650). The concentration of sOMV in the culture supernatants was calculated from a calibration curve which was based on the responses of the standards (sOMV corresponding with 0–2.5 mg/L total protein). In the changestat experiments, nanoparticle tracking analysis [59] was used for sOMV quantification. Static measurements (10 captures of 30 s) were made on a NanoSight NS500 with 488 nm laser module and sCMOS camera, that was calibrated with the concentration upgrade [60]. Temperature was controlled at 25 °C and captures were analyzed with the NTA 3.2 software build 3.2.16. Automated flow measurements were made as described previously [61].

OMV size was assessed by dynamic light scattering in a Zetasizer Nano-ZS with Zetasizer 7.11 software (Malvern Instruments). Measurements were performed using a SOP that takes three measurements in backscatter mode, with auto measurement duration and “seek for optimal position” as positioning setting. The sample was assumed to be protein with a refractive index of 1.450 and 0.001 absorption, in water as dispersant with a viscosity of 0.8872 cP and refractive index of 1.330. Data was processed with the normal analysis model.

### Sds-page

Purified OMVs were assessed for total protein content by the Lowry protein assay using Peterson’s modification. OMVs corresponding to 4 µg of protein were loaded on a precast polyacrylamide gel (Lonza) to perform SDS-gel electrophoresis. The gel was stained with InstantBlue protein stain (Expedeon).

## Additional file

**Additional file 1: Figure S1.** Increased dissolved oxygen tension triggers OMV release in *E. coli*. Changestat of *E. coli* (A) shows growth at dissolved oxygen tensions up to 200% air saturation in a changestat with a_DOT_ = 1.5%/h. OMV release is directly related to the increased oxygen concentration.

## Abbreviations

OMV: outer membrane vesicle
sOMV: spontaneously released OMV
eOMV: extracted OMV
dOMV: detergent extracted OMV
LPS: lipopolysaccharides
D: dilution rate
*a*_*D*_: acceleration rate of the dilution rate
*a*_*DOT*_: acceleration rate of the dissolved oxygen tension
CER: carbon dioxide evolution rate
OUR: oxygen uptake rate
RQ: respiratory quotient
DO: dissolved oxygen
ROS: reactive oxygen species
TP: total protein
NTA: nanoparticle tracking analysis
NET: neutrophil extracellular trap
Y_px_: OMV yield per biomass
Y_xs_: biomass yield on substrate
m_s_: maintenance coefficient
OD_590_: optical density at 590 nm
