# Supplementary figures and images for: High dissolved oxygen tension triggers outer membrane vesicle formation by Neisseria meningitidis
Source: Microb Cell Fact. 2018 Oct 3;17:157. doi: 10.1186/s12934-018-1007-7 (PMC6171317; doi:10.1186/s12934-018-1007-7)

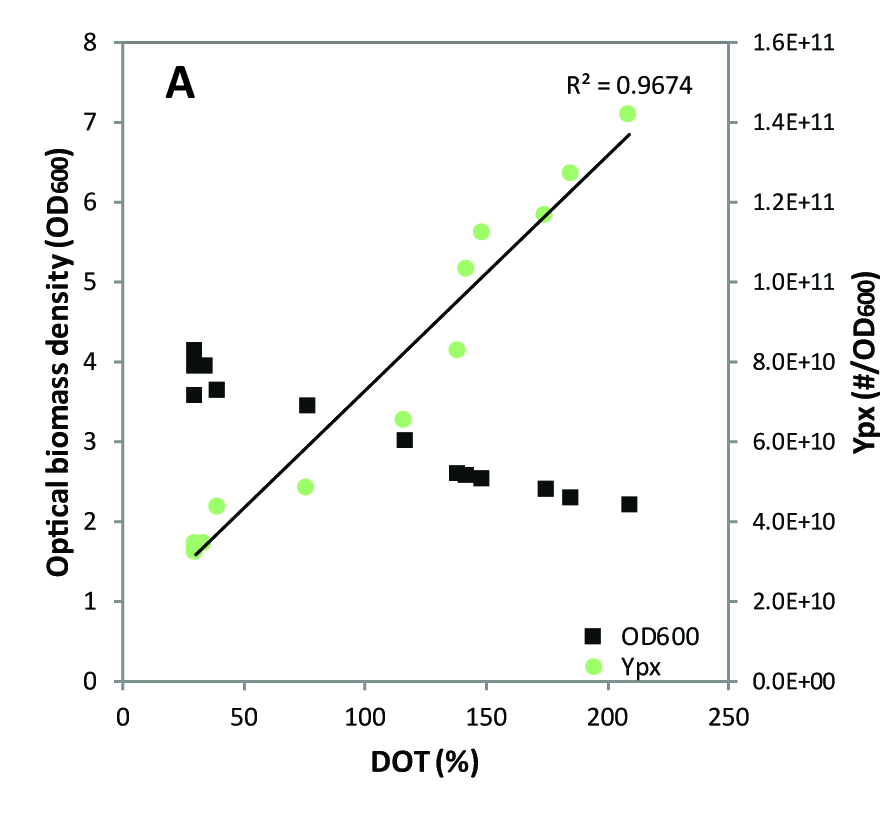

Supplement: Supplementary file 1 — Additional file 1: Figure S1. Increased dissolved oxygen tension triggers OMV release in E. coli. Changestat of E. coli (A) shows growth at dissolved oxygen tensions up to 200% air saturation in a changestat with aDOT = 1.5%/h. OMV release is directly related to the increased oxygen concentration. [file 12934_2018_1007_MOESM1_ESM.tif]
